# Transmission dynamics of low pathogenicity avian influenza (H2N2) viruses in live bird markets of the Northeast United States of America, 2013–2019

**DOI:** 10.1093/ve/veac009

**Published:** 2022-02-09

**Authors:** David H Chung, Mia. K Torchetti, Mary L Killian, David E Swayne, Dong-Hun Lee

**Affiliations:** US Department of Agriculture, National Veterinary Services Laboratories, Animal and Plant Health Inspection Service, 1920 Dayton Avenue, P.O. Box 844, Ames, IA 50010, USA; US Department of Agriculture, National Veterinary Services Laboratories, Animal and Plant Health Inspection Service, 1920 Dayton Avenue, P.O. Box 844, Ames, IA 50010, USA; US Department of Agriculture, Southeast Poultry Research Laboratory, US National Poultry Research Center, Agricultural Research Service, 934 College Station Road, Athens, GA 30605, USA; Department of Pathobiology and Veterinary Science, University of Connecticut, 61 North Eagleville Road, Unit-3089, Storrs, CT 06269, USA

**Keywords:** epidemiology, low pathogenicity avian influenza virus, H2N2, wild bird, poultry, phylogenetic analysis

## Abstract

Live bird market (LBM) surveillance was conducted in the Northeast United States (US) to monitor for the presence of avian influenza viruses (AIV) in domestic poultry and market environments. A total of 384 H2N2 low pathogenicity AIV (LPAIV) isolated from active surveillance efforts in the LBM system of New York, Connecticut, Rhode Island, New Jersey, Pennsylvania, and Maryland during 2013–2019 were included in this analysis. Comparative phylogenetic analysis showed that a wild-bird-origin H2N2 virus may have been introduced into the LBMs in Pennsylvania and independently evolved since March 2012 followed by spread to LBMs in New York City during late 2012–early 2013. LBMs in New York state played a key role in the maintenance and dissemination of the virus to LBMs in the Northeast US including reverse spread to Pennsylvania LBMs. The frequent detections in the domestic ducks and market environment with viral transmissions between birds and environment possibly led to viral adaptation and circulation in domestic gallinaceous poultry in LBMs, suggesting significant roles of domestic ducks and contaminated LBM environment as reservoirs in maintenance and dissemination of H2N2 LPAIV.

## 1. Introduction

Live bird markets (LBMs) are implicated as an optimal environment for avian influenza virus (AIV) maintenance, replication, and spread among susceptible poultry species co-housed with daily introduced naive poultry leading to viral maintenance within the market. The Northeast (NE) United States (US) has the largest number of LBMs in the USA with a complex system of production flocks, dealers/haulers, and markets (Garber et al. 2007; Jagne, Bennett, and Collins 2021). The LBMs in the NE US, consisting of more than 140 markets across 8 states [Pennsylvania (PA), New York (NY), New Jersey (NJ), Connecticut (CT), Rhode Island (RI), Maryland (MD), Massachusetts (MA), and New Hampshire (NH)], serve as a major source of fresh poultry meat products for mainly ethnic immigrant populations in large cities (Senne, Pedersen, and Panigrahy 2005). Avian influenza surveillance in these markets is voluntary under the ‘Prevention and Control of H5 and H7 Avian Influenza in the Live Bird Marketing System Uniform Standards for a State Federal-Industry Cooperative Program (USDA-APHIS 2020)’. As of 2021, the States of NY, PA, and NJ have the most active systems with NY State having the most markets presently at 87, followed by 36 in NJ, 11 in PA, and a small number (1–3) of LBMs in each of other states (Jagne, Bennett, and Collins 2021).

The frequent detections of AIV from LBMs raised public health concerns. The NE US LBMs were identified as the probable source of the virus responsible for the Pennsylvania H5N2 low pathogenic AIVs (LPAIVs) outbreak in the summer of 1983 which subsequently mutated to high pathogenicity (HP) AIV in October 1983 resulting in the HPAIV outbreak in 1983–4 in PA, VA, and MD (Senne, Pedersen, and Panigrahy 2005). During 1996–2006, the H7N2 LPAIVs spread from LBMs of the NE US causing five consecutive outbreaks in commercial poultry despite the efforts to eradicate the virus by market closures followed by extensive cleaning and disinfection (Akey 2003; Davison, Eckroade, and Ziegler 2003; Dunn et al. 2003). The voluntary LBM surveillance program was established in NE US LBMs to monitor AIVs after the H5N2 virus outbreak in PA during the mid-1980s (Senne, Pedersen, and Panigrahy 2005). The NE US LBMs have been inspected and tested at least once per quarter, including testing of AIV from five to eleven randomly selected birds of each bird type and environmental samples from each market (Jagne, Bennett, and Collins 2021).

The H2N2 subtype has been sporadically detected in NE US LBMs (Schafer et al. 1993; Joseph et al. 2015). Previous studies demonstrated that H2N2 viruses detected in the 1990s formed a monophyletic clade, indicating that the viruses were closely related and shared a common ancestry. This current LBM H2N2 lineage is neither related to the clade from 1990 nor are they related to the human pandemic 1957 H2N2 influenza A virus, which belong to a Eurasian lineage and demonstrated a low or intermediate risk to mammalian transmissions (Schafer et al. 1993; Makarova et al. 1999; Liu et al. 2004; Piaggio et al. 2012; Jones et al. 2014). In this study, 384 H2N2 LPAIV submitted to the United States Department of Agriculture’s (USDA) Animal and Plant Health Inspection Services (APHIS) from active voluntary surveillance efforts at the NE US LBMs, including NY, CT, RI, NJ, PA, and MD during 2013–2019 were sequenced and analyzed to aid in tracing the origin and investigate the transmission dynamics by incorporating host species and sampling locations into statistical Bayesian phylogenetic analysis models.

## 2. Methods

### 2.1 Genome sequencing

A total of 431 H2N2 LPAIV collected from LBMs in the NE US and confirmed by the USDA APHIS during 2013–2019 were sequenced in this study. We excluded low coverage/poor quality data and obtained complete genome sequences of 381 viruses, including 288 from NY, 57 from NJ, 17 from CT, 14 from PA, 3 from RI, and 2 from MD. All eight segments of the isolates were amplified by multiplex RT-PCR and whole-genome sequenced by using the MiSeq system (Illumina, San Diego, CA, USA) as previously described (Lee 2020). Briefly, the Nextera XT DNA Sample Preparation Kit (Illumina) and the 500 cycle MiSeq Reagent Kit v2 (Illumina) were used according to the manufacturer’s instructions to generate and sequence multiplexed paired-end sequencing libraries. Genome sequence assembly was carried out using IRMA v.0.6.7 (Shepard et al. 2016) and verified manually using reference-based assembly in SeqMan NGen v14 program (http://www.dnastar.com/t-nextgen-seqman-ngen.aspx). Genome sequences were deposited in NCBI BioProject (BioProject accession code: PRJNA750416) and GenBank (accession nos. KY272856-KY272863, KY272971-KY272986, MW727341-MW727380, and MN998501-MN998508).

### 2.2 Intravenous pathogenicity index in chickens

The *in vivo* lethality testing for 34 representative viruses was conducted according to OIE (http://www.oie.int/en/international-standard-setting/terrestrial-manual). The selected representative samples include the index detection from each state and an additional virus newly detected every 6 months, or virus possessing any change in the HA cleavage site sequences. Briefly, 0.2 ml of a 1/10 dilution of infectious allantoic fluid was inoculated intravenously into eight 4- to 8-week-old specific-pathogen-free chickens and chickens monitored for 10 days for mortality; death in 6 to 8 birds is considered highly pathogenic. The *in vivo* pathogenicity assays with live viruses were conducted at the National Veterinary Services Laboratories, Animal and Plant Health Inspection Service, US Department of Agriculture in Ames, Iowa, USA, and in accordance with approved institutional animal care and use protocols.

### 2.3 Phylogenetic analysis

Comparative phylogenetic analysis was conducted to trace the evolution and spread of the H2N2 viruses in NE US. All closely related HA sequences of wild-bird origin H2 viruses searched by the Basic Local Alignment Search Tool (BLAST) search on Global Initiative on Sharing All Influenza Data (GISAID) database were added to the dataset as reference sequences which were exclusively wild-bird-origin H2 virus sequences. Reference sequences for phylogenetic analyses of PB2, PB1, PA, NP, NA, M, and NS gene segments were selected by BLAST search on GISAID database and subsampled based on nucleotide sequence identity of 99–99.5 per cent using CD-HIT (Fu et al. 2012) in February 2020. We generated maximum-likelihood phylogenies of each gene segment (PB2, ntax = 419; PB1, ntax = 429; PA, ntax = 435; HA, ntax = 411; NP, ntax = 440; NA, ntax = 423; M, ntax = 439; NS, ntax = 408) using RAxML v8 and the GTR nucleotide substitution model, with among-site rate variation modeled by using a discrete gamma distribution (Stamatakis 2014). Bootstrap support values were generated using 1,000 rapid bootstrap replicates. We performed a root-to-tip regression analysis against sampling dates on the ML tree using TempEst (http://tree.bio.ed.ac.uk/software/tempest/) to determine the temporal signal of the HA sequences. As this revealed significant clock-likeness, we performed two distinct Bayesian discrete trait phylogeographic analyses (DTA) using BEAST version 1.10.4 (Drummond and Rambaut 2007). (i) To investigate virus transmission between locations, we reconstructed the virus transmission history between states geographically using an ancestral state reconstruction approach with a Bayesian stochastic search variable selection to determine the most probable spatial transmission history. The closely related HA sequences of wild-bird origin H2 viruses from BLAST search added to the dataset were labeled as ‘Wild Bird’. In this analysis, we defined geographic region as discrete nominal categories, including Wild Bird (13 sequences), ‘New York’ (NY) (35 sequences), ‘Pennsylvania’ (PA) (14 sequences), ‘New Jersey’ (NJ) (29 sequences), and ‘Connecticut and Rhode Island’ (CT_RI) (20 sequences). To account for potential sampling biases, sequences were subsampled to preserve diversity with respect to location (state) which resulted in 111 sequences. Particularly, the NY and NJ dataset was subsampled based on nucleotide identity at 99.5 per cent and 99.8 per cent using CD-HIT (Huang et al. 2010), respectively. (ii) The phylogenetic data were also used to infer the transmission dynamics between host species in NY category, including ‘domestic Anseriformes in New York’ (Dm_Ans_NY) (103 sequences), ‘domestic Galliformes in New York’ (Dm_Gal_NY) (105 sequences), ‘environment in New York’ (Env_NY) (80 sequences), thus providing evidence for which host type plays a major role in the spread of H2N2 LPAIV in LBMs. The environmental samples include viruses detected in swabs from floors, cages, drains, walls, scales, door handles, etc. at LBMs. For both analyses, Bayesian relaxed clock phylogeny of HA gene was reconstructed by using BEAST version 1.10.4 (Drummond and Rambaut 2007). The HKY + G nucleotide substitution model with an uncorrelated lognormal relaxed molecular clock was used along with a Gaussian Markov Random Field (GMRF) Bayesian skyride coalescent tree prior (Minin, Bloomquist, and Suchard 2008). Bayes factor (BF) was calculated using the SPREAD v. 1.0.7 (Bielejec et al. 2011) to identify the best supported viral transitions between discrete categories. We identified a transition as significant when posterior probability >0.5 and BF >3. The rate and number of viral transitions between discrete categories (Markov jump) and the time spent in a state between transitions (Markov reward) were estimated using stochastic mapping (Minin and Suchard 2008). The Markov Chain Monte Carlo (MCMC) was run in parallel for 3 chains, each with 40–100 million steps and samples across chains combined after 10 per cent burn-in. The parameters were analyzed with TRACER v1.5 (http://tree.bio.ed.ac.uk/software/tracer/) and all parameters had an effective sample size greater than 200. A maximum clade credibility (MCC) tree was generated using TreeAnnotator and visualized using FigTree 1.4.4 (http://tree.bio.ed.ac.uk/software/figtree/). We analyzed posterior trees using the program PACT (http://www.trevorbedford.com/pact) to compute the number of transition events through time (0.25 years per section) and discrete state proportion through time.

To assess the presence of sampling bias in our analysis, we conducted the generalized linear model (GLM) analysis as an extension of DTA to identify the impact of viral source/sink sample sizes on our Bayesian discrete inference datasets using BEAUti v1.10.4 software (Drummond and Rambaut 2007). The GLM result indicates virus migration rates as a linear combination of coefficients and coefficient indicators, and predictors. The coefficient represents the effect size of predictors affecting the migration rate of the virus and the coefficient indicator describes if the predictor was included in the model. The sample size of each discrete state was used as a predictor to inform the viral transition rates of two separate DTA.

## 3. Results

**Figure 1.**
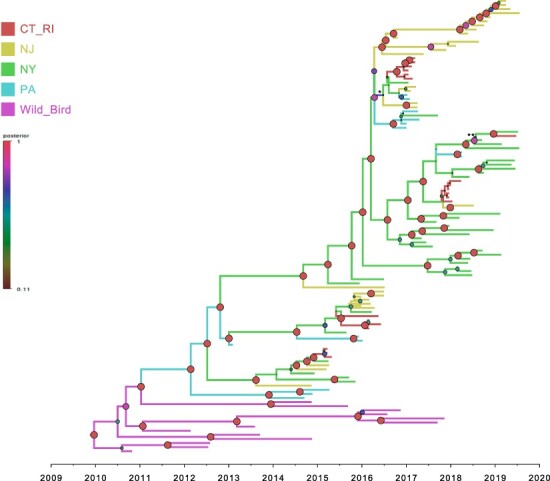
Bayesian phylogenetic tree of hemagglutinin gene of H2N2 viruses. The geographic regions were defined in the analysis as discrete states, including ‘Connecticut and Rhode-Island’ (CT_RI), ‘New Jersey’ (NJ), ‘New York’ (NY), ‘Pennsylvania’ (PA), and ‘wild bird.’ Branches are colored according to geographic region. Posterior probabilities of nodes are indicated by the size and color of circles at each node. The branches assigned with uncertain discrete traits were shown with asterisks (*NY and NJ, **NY and CT_RI).

Lethality testing of representative LBM isolates confirmed that the H2N2 isolates were of low pathogenicity (no mortalities). ML phylogenetic analysis for each of the eight gene segments was used to infer the reassortment events of H2N2 LPAIV (Fig. S1). Each gene segment exhibited a well-supported monophyletic clade of H2N2 LPAIV, suggesting the H2N2 LPAIV evolved in the absence of reassortment during 2013–2019. The H2N2 viruses detected in the 1990s were not phylogenetically related to the currently circulating H2N2 viruses in the NE US (data not shown). Molecular dating analysis of HA gene demonstrated that the time to most recent common ancestor (TMRCA) of H2N2 LPAIV in NE US LBMs was estimated to be 1 March 2012 [95 per cent Bayesian credible interval (BCI): 26 September 2011–27 June 2012] (Fig. 1 and S2). The mean substitution rate of HA gene was 6.617 × 10^−3^ substitutions/site/year (95 per cent BCI: 4.67–8.80 × 10^−3^), which is in range of global AI virus substitution rates as described elsewhere (Chen and Holmes 2006).

**Figure 2.**
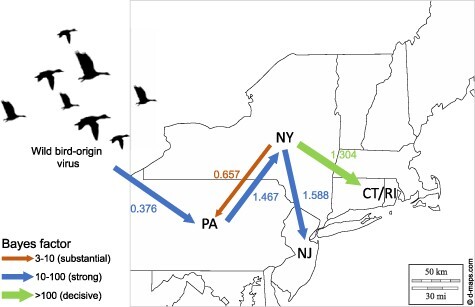
Inferred transmission networks of H2N2 viruses between live bird markets in the NE US. Arrows show the direction of transmission. Line colors indicate the overall Bayes Factor test support for epidemiological linkage. Green lines indicate statistical support with BF > 100 (very strong support), Blue lines indicate support with 10 < BF < 100 (strong support), and red lines indicate support with 3 < BF < 10. Numbers next to arrows indicate transition rates.

**Figure 3.**
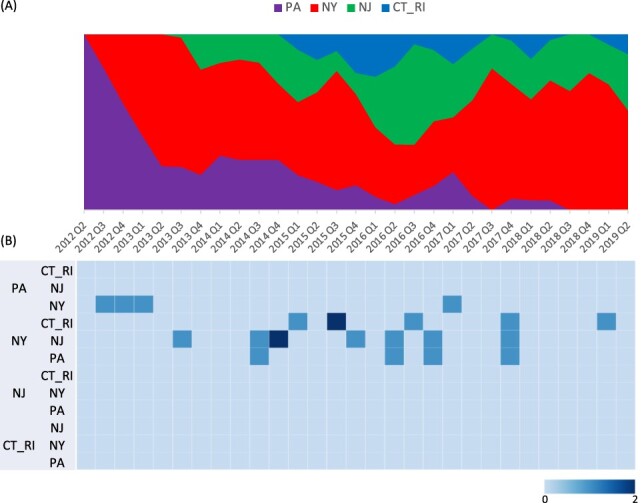
(A) Proportion of ancestral discrete states estimated on the phylogenetic trunk of H2N2 viruses in NE US LBMs through time. The geographic region of LBM was defined as discrete states, including ‘Pennsylvania’ (PA), ‘New York’ (NY), ‘New Jersey’ (NJ), and Connecticut and Rhode Island’ (CT_RI). Shaded areas represent estimated ancestral discrete state proportions of each state. At each point in time, the width (*y*-axis) represents the mean proportion from 0 to 100 per cent of each state. (B) Heat map showing the number of transition events between each discrete state (0–2).

**Figure 4.**
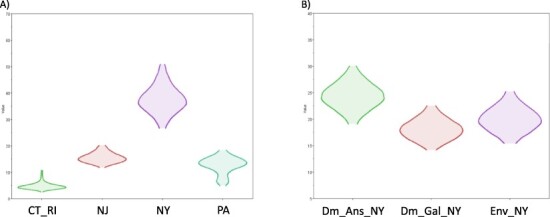
Density distribution of the total time spent in each discrete state category (Markov rewards). The geographic region of LBM was defined as discrete states, including ‘Pennsylvania’ (PA), ‘New York’ (NY), ‘New Jersey’ (NJ), and Connecticut and Rhode Island’ (CT_RI). (A) Markov rewards of CT_RI (5.190, 95 per cent HPD: 2.535–9.889), NJ (15.751, 95 per cent HPD: 11.902–20.508), NY (37.550, 95 per cent HPD: 26.575–50.236), PA (12.1960, 95 per cent HPD: 4.947–18.607), and Wild_Bird (26.203, 95 per cent HPD: 21.004–31.621) were calculated for each posterior tree in non‐reversible continuous-time Markov chain model. (B) Density distribution of the total time spent in a particular host population in New York (Markov rewards). Markov rewards of Dm_Ans_NY (24.324 95 per cent HPD: 18.722–29.706), Dm_Gal_NY (18.178, 95 per cent HPD: 14.142–22.603), Env_NY (19.993, 95 per cent HPD: 15.120–25.307) were calculated for each posterior tree in non‐reversible continuous‐time Markov chain model.

Source sink dynamics of the H2N2 LPAIV identified from the LBMs in NE US during 2013–2019 was analyzed by estimating the transition rate (TR), Markov jump count, and Markov rewards. The GLM analysis revealed the potential impact of sample size has negligible influence to cause a potential bias toward the origin or destination state in this analysis (source indicator: 0.391, source coefficient: 0.419, sink indicator: 0.416, and sink coefficient: 0.251) (Table 1). Bayesian phylodynamic simulation exhibited that H2N2 LPAIV was most likely introduced from wild birds into domestic flocks in Pennsylvanian (PA) LBMs with significant statistical support (posterior probability >0.5 and BF >3; Figs 1 and 2). Based on our DTA, well-supported viral transmission with introduction from Wild_Bird to PA LBM (TR: 0.376, BF: 14.463, posterior probability: 0.816) and onward spread from PA LBM to NY LBM (TR: 1.467 BF: 41.929, posterior probability: 0.928) occurred (Fig. 2 and Table 2). The well-supported transitions from NY to PA (TR: 1.467, BF: 41.929, posterior probability: 0.928), NY to NJ (TR: 1.588, BF: 45.941, posterior probability: 0.934), and NY to CT_RI (TR: 1.304, BF: 118.497, posterior probability: 0.973) suggest the subsequent spread of the viruses from NY LBM to neighboring states including reverse spread to PA LBM after the multiple virus introductions from PA LBM to NY LBM in late 2012 and early 2013 (Fig. 3). The total Markov reward time was the highest in LBMs in NY (37.550, 95 per cent HPD: 26.575–50.236), followed by NJ (15.751, 95 per cent HPD: 11.902–20.508), PA (12.1960, 95 per cent HPD: 4.947–18.607), and CT_RI (5.190, 95 per cent HPD: 2.535–9.889) (Fig. 4A). Collectively, these results indicate that the LBMs in NY play a central role in the maintenance and spread of H2N2 in NE US.

**Table 1.** The source/sink indicator and coefficient values of GLM analyses using samples sizes of each discrete state in DTA between NE US LBMs and host species in NY LBMs

Discrete States	Source indicator1	Sink coefficient1	Source indicator2	Sink coefficient2
LBM location	0.391	0.419	0.416	0.251
Host species in NY LBMs	0.163	−0.088	0.033	0.005

**Table 2.** The well-supported transition rate, Bayes Factor, and statistical support value of discrete trait phylodynamic analysis between LBMs in Northeastern states, United States.

Transition from	Transition to	Mean actual migration rate^a^ (95% BCI^b^)	Bayes Factor	Posterior probability
Wild_Bird	PA^c^	0.376 (0–1.136)	14.463	0.816
PA	NY^d^	1.467 (0–3.347)	41.929	0.928
NY	PA^c^	0.657 (0–7.370)	6.647	0.671
NY	NJ^e^	1.588 (0–3.301)	45.941	0.934
NY	CT_RI^f^	1.304 (0–2.855)	118.497	0.973

a Actual migration rates were calculated as the rate × indicator.

b BCI: Bayesian credibility interval.

c PA: Pennsylvania,

d NY: New York,

e NJ: New Jersy, and

^f^CT_RI: Connecticut and Rhode Island.

**Figure 5.**
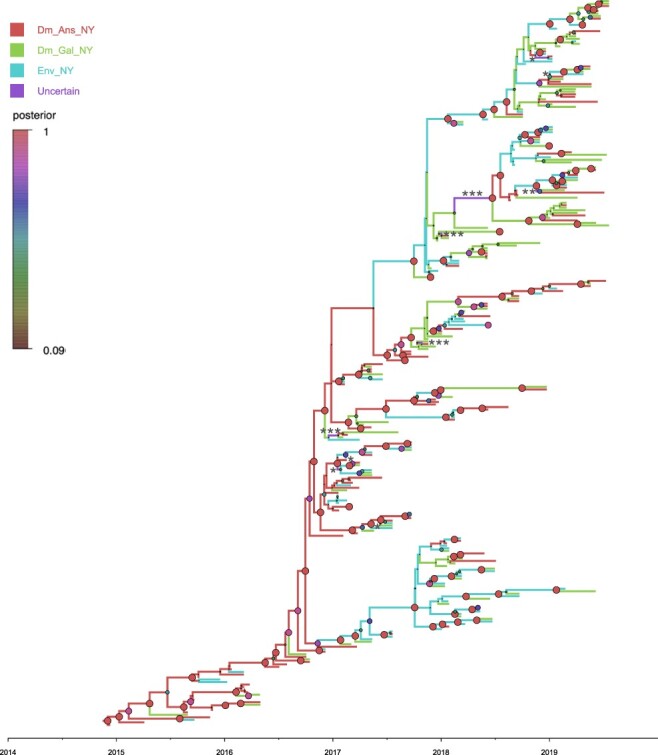
Bayesian phylogenetic tree of hemagglutinin gene of H2N2 viruses identified in the LBMs in New York. The host type was defined in the analysis as discrete states, including ‘domestic Anseriformes in LBMs, New York’ (Dm_Ans_NY), ‘domestic Galliformes in LBMs, New York’ (Dm_Gal_NY), and ‘environmental samples from LBMs in New York’ (Env_NY). Branches are colored according to host type. Posterior probabilities of nodes are indicated by the size and color of circles at each node. The branches assigned with uncertain discrete traits were shown with asterisks (*Dm_Ans_NY and Env_NY, **Dm_Gal_NY and Env_NY, ***Dm_Ans_NY and Dm_Gal_NY).

**Figure 6.**
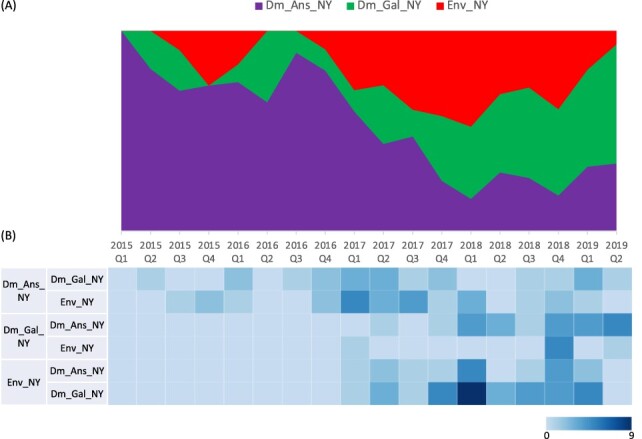
(A) Proportion of ancestral discrete states estimated on the phylogenetic trunk of H2N2 viruses in NY LBMs through time. The host type was defined as discrete states in the Bayesian phylogenetic analysis, including ‘domestic Anseriformes in New York’ (Dm_Ans_NY), ‘domestic Galliformes in New York’ (Dm_Gal_NY), ‘environment in New York’. Shaded areas represent estimated ancestral discrete state proportions of each state. At each point in time, the width (*y*-axis) represents the mean proportion from 0 to 100 per cent of each state. (B) Heat map showing the number of transition events between each discrete state (0–9).

As a secondary analysis to explore the extent of viral maintenance and spread between host species and environment within LBMs in NY, DTA was conducted with three discrete nominal categories: Dm_Ans_NY, Dm_Gal_NY, and Env_NY. The frequent detections of virus transitions from Env_NY to Dm_Ans_NY (TR: 0.941, BF: 2616.411, posterior probability: 1.000), Env_NY to Dm_Gal_NY (TR: 1.471, BF: 49733.920, posterior probability: 1.000), Dm_Ans_NY to Env_NY (TR: 0.980, BF: 4972.287, posterior probability: 1.000), Dm_Ans_NY to Dm_Gal_NY (TR: 0.873, BF: 4520.149, posterior probability: 1.000), and Dm_Gal_NY to Dm_Ans_NY (TR: 1.023, BF: 49733.920, posterior probability: 1.000), suggest the H2N2 LPAIV is frequently transmitted between poultry and environment, and maintained in Anseriformes poultry and environment of NY LBMs (Fig. 5 and Table 3). The GLM analysis revealed the potential impact of sample size is negligible in this analysis (source indicator: 0.163, source coefficient: −0.088, sink indicator: 0.033, and sink coefficient: 0.005; Table 1). The Dm_Ans_NY exhibited the highest Markov reward time (24.324, 95 per cent HPD: 18.722–29.706) followed by Env_NY (19.993, 95 per cent HPD: 15.120–25.307), and Dm_Gal_NY (18.178, 95 per cent HPD: 14.142–22.603; Fig. 4B). The Markov reward time data are also consistent with previous findings on the significant role of domestic ducks in LBMs to facilitate the introduction and transmission of AIVs (Kwon et al. 2020; Youk et al. 2020). Frequent viral transmissions from Dm_Ans_NY to Dm_Gal_NY and Env_NY support the idea that domestic ducks may have acted as a seeding population in the LBMs in NY that is concordant with the highest trunk proportion being identified in Dm_Ans_NY during 2015–2016 (Fig. 6). An underlying mechanism of dynamics of circulating H2N2 LPAIV could be due to the cycle of deposition of viral pathogens in contaminated environment and reintroduction of the virus into newly introduced naïve poultry in the market system (Aguero-Rosenfeld et al. 2005). Our data highlight the key role of domestic ducks and environmental virus depositions in the persistence of H2N2 LPAIV in LBMs in NY, followed by dissemination to LBMs in neighboring states.

**Table 3.** The well-supported transition rate, Bayes Factor and statistical support value of discrete trait phylodynamic analysis between host species and environment in LBMs in the State of New York, United States.

Transition from	Transition to	Mean actual migration rate^a^ (95% BCI^b^)	Bayes Factor	Posterior probability
Dm_Ans_NY^c^	Dm_Gal_NY^d^	0.873 (0.122-1.815)	4520.149	1.000
Dm_Ans_NY	Env_NY^e^	0.980 (0.190–1.941)	4972.287	1.000
Dm_Gal_NY	Dm_Ans_NY	1.023 (0.163–2.073)	49,733.920	1.000
Env_NY	Dm_Ans_NY	0.941 (0.151–1.977)	2616.411	1.000
Env_NY	Dm_Gal_NY	1.471 (0.300–2.842)	49,733.920	1.000

a Actual migration rates were calculated as the rate × indicator.

b BCI: Bayesian credibility interval.

c Dm_Ans_NY: Domestic Anseriformes species in LBMs, New York,

d Dm_Gal_NY: Galliformes species in LBMs, New York,

and ^e^ENV_NY: Environment samples from LBMs in New York.

We identified the NA stalk region deletions in 3 different clades designated as subgroup A which contains 3 NJ viruses collected in 2016, subgroup B which contains 30 viruses collected in NJ 2018–2019, and subgroup C which contains 4 NY viruses collected in 2019 (Fig. S1). The sequences in subgroup A had distinct NA stalk deletion at amino acid positions of 56–72 (A/Ck/NJ/16-007567-007), 51–72(A/Ck/NJ/16-021454-003), and 44–65 (A/guinea_fowl/NJ/16-021454-002). All 34 sequences in subgroups B and C exhibited NA stalk deletion at amino acid positions of 62–83. In particular, 31 samples were isolated from gallinaceous poultry species including chicken, guinea fowl, quail, and chukar, while 5 samples were from Anseriformes (Muscovy ducks) and 1 sample was from the LBM environment. The NA stalk region deletions may have been associated with host adaptation of H2N2 LPAIV in gallinaceous poultry in LBMs (Castrucci and Kawaoka 1993; Guangxiang, Jeffrey, and Palese 1993).

## 4. Discussion

The risk of AIV influx and spread within the LBM system is constantly present due to the large avian population from various sources residing in the markets (Cardona, Yee, and Carpenter 2009; Jagne, Bennett, and Collins 2021). In the NE US live bird marketing system, poultry are raised on special production farms or commercial farms that sell wholesale to dealers and haulers who in turn provide to retail LBMs (Jagne, Bennett, and Collins 2021). In particular, poultry producers from PA provide the majority of the birds going into the LBMs for all the NE States. Our phylogeography data coincided with this poultry production and supply flow supporting that the initially introduced H2N2 LPAIV to PA subsequently spread to NY which has the largest number of LBMs in the US. After the multiple virus introductions from PA to NY LBMs in late 2012 and early 2013, the H2N2 viruses appear to have been maintained in NY LBMs and frequently spread to neighboring states including reverse spread to PA LBM. We were not able to investigate the association between interstate poultry trade and its role on the spread of H2N2 viruses due to limited information for poultry trade and supply networks between LBMs. The H2N2 virus in the LBMs has independently evolved since March 2012 with the same gene constellation until mid-2019. During the preparation of this article, we detected reassortant viruses in NY LBM possessing PB2, PB1, PA, and NS genes from other North American lineage non-H2N2 wild-bird-origin viruses in late 2019 (data not shown). Ongoing surveillance and sequencing will aid in determining whether these represent transient or may become established in LBMs.

In the present study, two separate phylodynamic analyses were used to estimate the most probable transmission dynamics between the LBMs in the NE states of USA, and between host species and environment within the NY LBMs. Our analysis between locations provides strong evidence for a central role of NY LBMs in the maintenance, amplification, and spread of H2N2 viruses via LBM chains from late 2013 to early 2019. It should be noted that we used only HA gene for this analysis, but transmission dynamics of other genes were not investigated. Although all eight genes of viruses in our dataset were monophyletic in ML analysis, there is a possibility that minor reassortment events occurred between the H2N2 viruses which could possibly cause alterations in the tree topology of other genes and influence the posterior probability of NY state as a source location. The secondary DTA was conducted to further investigate the transmission history of H2N2 between host species and the environment within NY LBMs. This analysis highlights the key role of domestic ducks and environmental virus depositions in persistence of H2N2 LPAIV in LBMs in NY, followed by dissemination to LBMs in neighboring states. We used all viruses identified from NY LBMs in the analysis since the NY LBMs acted only as a source population after the initial virus introductions from PA LBM during 2012–2013. It is most likely that there was no virus influx from other states to NY LBM except only one case from PA to NY (A/Muscovy_duck/NY/17-031928-003/2017) during the first quarter of 2017 as shown in Fig. 3. The transmitted A/Muscovy_duck/NY/17-031928-003/2017 virus was negligible in the DTA model because it disappeared without further circulations in NY LBMs.

The dissemination of H2N2 LPAIV between LBMs was most likely promoted by the trading of birds via market chains. In the trading process, flocks are collected at the farms or LBMs and distributed via ground transportation to distribution centers or directly to LBMs (Senne, Pedersen, and Panigrahy 2005). Newly entering birds in the LBMs originate from various sources including commercial poultry farms and backyard flocks in neighboring states. New birds are introduced to the LBMs daily and co-housed or caged closely with precedent flocks providing optimal conditions for AIVs to replicate and adapt to the new hosts which may result in long-term viral maintenance. The infectious period of AIV in infected birds is well-established to range from 6 to 10 days (Stallknecht et al. 1990; Webster et al. 1992; Brown et al. 2006). During the period, infected avian hosts undergo continuous shedding of virions in their feces and respiratory secretions which may transmit the virus directly to other hosts by ingestion or inhalation or may lead to limited viral maintenance in the environment. The viral shedding of infected poultry can contaminate the LBM environment samples, including swabs from floors, cages, drains, walls, scales, and door handles, from which the virus may persist for days or weeks depending on the temperature and other environmental factors, and reinfect host avian species (Breban et al. 2009; Jagne, Bennett, and Collins 2021). Based on our analysis, the frequent H2N2 AIV detections in the poultry and waterfowl, as well as the market environments emphasize the potential role of H2N2 LPAIV contamination in the LBM environment as an ongoing source of infection for incoming naïve domestic poultry. The H2N2 LPAIV Markov reward time and transmission data between discrete host categories in the NY LBMs indicate extensive viral maintenance within domestic Anseriformes and in the LBM environments which could facilitate viral spread between the susceptible hosts. Consistent with this finding, housing susceptible poultry species with ducks in proximity was considered as a potential risk in NE US LBMs due to characteristics of ducks as a natural AIV reservoir (Webster et al. 1992). Previous surveillance studies in East Asian LBMs also suggested the domestic duck as a major contributor to the spread and maintenance of various AIV strains (Mase et al. 2005; Wee et al. 2006; Uchida et al. 2008; Kim et al. 2010, 2012; Sakoda et al. 2012; Lee et al. 2014; Kanehira et al. 2015; Kwon et al. 2016, 2017, 2020).

Long-term circulation of wild-bird-origin AIVs in LBMs allows viruses to adapt among domestic poultry affecting viral host specificity and/or virulence via genetic evolution. Due to the confined spaces in LBM settings, different avian species could be caged in the same market with close proximity and it was commonly practiced in LBMs before regulations, such as Uniform Standard Program, were enacted (Jagne, Bennett, and Collins 2021). Since waterfowl are the natural reservoir for AIVs, viral transmission risks are present from co-housing domestic Gallinaceous species and domestic waterfowl population from various sources. In previous studies, changes in the viral host range were related to the length of NA stalk region of AIVs (Castrucci and Kawaoka 1993; Guangxiang, Jeffrey, and Palese 1993). Truncation in NA stalk region of AIVs has been associated with viral host adaptation in gallinaceous species (Banks et al. 2001; Hossain, Hickman, and Perez 2008; Sorrell et al. 2010). The majority of the domestic host species affected by viruses with NA truncation have been domestic gallinaceous poultry. In the current study, the presence of multiple NA stalk region deletions supports adaptation, extensive transmission, and long-term circulation of H2N2 LPAIV, not only in domestic waterfowl but also in gallinaceous poultry in the LBMs.

The complete genome sequences produced in this study greatly expanded the existing data set of AIV genome sequences from LBMs in the USA and allowed us to determine the evolutionary history and molecular epidemiology of H2N2 LPAIVs detected in LBMs across spatial and temporal scales. Molecular analysis demonstrated that the current LBM lineage H2N2 LPAIVs that has circulated in the NE US LBMs since 2012 continues to evolve, has acquired markers of gallinaceous poultry adaptation as early as 2019, and has reassorted with wild bird lineage viruses on more than one occasion. In addition, transmission and maintenance of the virus in domestic ducks and the LBM environment were important epidemiological features of the H2N2 LPAIV. These results highlight the potential for domestic ducks and environmental contamination in the LBMs to serve as reservoirs to maintain and disseminate AIV, subsequently contributing to viral adaptation and circulation in domestic poultry. Coordinated response measures such as trace outs, and heightened biosecurity measures such as market sell-downs, closures for cleaning and disinfection have been recommended by a subcommittee of the Live Bird Marketing Working Group that was created to help address this issue. Continued genomic surveillance is one tool in aiding the eradication of this virus and is crucial for ongoing monitoring of AIV incursions into the NE US LBMs.

## Supplementary Material

veac009_Supp

## References

[R1] Aguero-Rosenfeld M. E. et al. (2005) ‘Diagnosis of Lyme Borreliosis’, *Clinical Microbiology Reviews*, 18: 484–509. 16020686 10.1128/CMR.18.3.484-509.2005 PMC1195970

[R2] Akey B. L. (2003) ‘Low-pathogenicity H7N2 Avian Influenza Outbreak in Virgnia during 2002’, *Avian Diseases*, 47: 1099–103. 14575120 10.1637/0005-2086-47.s3.1099

[R3] Banks J. et al. (2001) ‘Changes in the Haemagglutinin and the Neuraminidase Genes Prior to the Emergence of Highly Pathogenic H7N1 Avian Influenza Viruses in Italy’, *Archives of Virology*, 146: 963–73. 11448033 10.1007/s007050170128

[R4] Bielejec F. et al. (2011) ‘SPREAD: Spatial Phylogenetic Reconstruction of Evolutionary Dynamics’, *Bioinformatics*, 27: 2910–2. 21911333 10.1093/bioinformatics/btr481 PMC3187652

[R5] Breban R. et al. (2009) ‘The Role of Environmental Transmission In Recurrent Avian Influenza Epidemics’, *PLoS Computational Biology*, 5: e1000346. 10.1371/journal.pcbi.1000346 PMC2660440 19360126

[R6] Brown J. D. et al. (2006) ‘Susceptibility of North American Ducks and Gulls to H5N1 Highly Pathogenic Avian Influenza Viruses’, *Emerging Infectious Diseases*, 12: 1663–70. 17283615 10.3201/eid1211.060652 PMC3372354

[R7] Cardona C., Yee K., and Carpenter T. (2009) ‘Are Live Bird Markets Reservoirs of Avian Influenza?’ *Poultry Science*, 88: 856–9. 10.3382/ps.2008-00338 19276435

[R8] Castrucci M. R., and Kawaoka Y. (1993) ‘Biologic Importance of Neuraminidase Stalk Length in Influenza A Virus’, *Journal of Virology*, 67: 759–64. 8419645 10.1128/jvi.67.2.759-764.1993 PMC237428

[R9] Chen R., and Holmes E. C. (2006) ‘Avian Influenza Virus Exhibits Rapid Evolutionary Dynamics’, *Molecular Biology and Evolution*, 23: 2336–41. 16945980 10.1093/molbev/msl102

[R10] Davison S., Eckroade R. J., and Ziegler A. F. (2003) ‘A Review of the 1996-98 Nonpathogenic H7N2 Avian Influenza Outbreak in Pennsylvania’, *Avian Diseases*, 47: 823–7. 14575071 10.1637/0005-2086-47.s3.823

[R11] Drummond A. J., and Rambaut A. (2007) ‘BEAST: Bayesian Evolutionary Analysis by Sampling Trees’, *BMC Evolutionary Biology*, 7. 10.1186/1471-2148-7-214 PMC2247476 17996036

[R12] Dunn P. A. et al. (2003) ‘Summary of the 2001-02 Pennsylvania H7N2 Low Pathogenicity Avian Influenza Outbreak in Meat Type Chickens’, *Avian Diseases*, 47: 812–6. 14575069 10.1637/0005-2086-47.s3.812

[R13] Fu L. et al. (2012) ‘CD-HIT: Accelerated for Clustering the Next-generation Sequencing Data’, *Bioinformatics*, 28: 3150–2. 23060610 10.1093/bioinformatics/bts565 PMC3516142

[R14] Garber L. et al. (2007) ‘Description of Live Poultry Markets in the United States and Factors Associated with Repeated Presence of H5/H7 Low-pathogenicity Avian Influenza Virus’, *Avian Diseases*, 51: 417–20. 17494597 10.1637/7571-033106R.1

[R15] Guangxiang L., Jeffrey C., and Palese P. (1993) ‘Alterations of the Stalk of the Influenza Virus Neuraminidase: Deletions and Insertions’, *Virus Research*, 29: 141–53. 8212856 10.1016/0168-1702(93)90055-r

[R16] Hossain M. J., Hickman D., and Perez D. R. (2008) ‘Evidence of Expanded Host Range and Mammalian-associated Genetic Changes in a Duck H9N2 Influenza Virus following Adaptation in Quail and Chickens’, *PLoS One*, 3: e3170. 10.1371/journal.pone.0003170 PMC2525835 18779858

[R17] Huang Y. et al. (2010) ‘CD-HIT Suite: A Web Server for Clustering and Comparing Biological Sequences’, *Bioinformatics*, 26: 680–2. 20053844 10.1093/bioinformatics/btq003 PMC2828112

[R18] Jagne J. F., Bennett J., and Collins E. (2021) ‘Live Bird Markets of the Northeastern United States’, *Delaware Journal of**Public Health*, 7: 52–6. 10.32481/djph.2021.01.009 PMC8352538 34467179

[R19] Jones J. C. et al. (2014) ‘Risk Assessment of H2N2 Influenza Viruses from the Avian Reservoir’, *Journal of Virology*, 88: 1175–88. 24227848 10.1128/JVI.02526-13 PMC3911670

[R20] Joseph U. et al. (2015) ‘Adaptation of Pandemic H2N2 Influenza A Viruses in Humans’, *Journal of Virology*, 89: 2442–7. 25505070 10.1128/JVI.02590-14 PMC4338906

[R21] Kanehira K. et al. (2015) ‘Characterization of an H5N8 Influenza A Virus Isolated from Chickens during an Outbreak of Severe Avian Influenza in Japan in April 2014’, *Archives of Virology*, 160: 1629–43. 25902725 10.1007/s00705-015-2428-9

[R22] Kim H. R. et al. (2012) ‘Highly Pathogenic Avian Influenza (H5N1) Outbreaks in Wild Birds and Poultry, South Korea’, *Emerging Infectious Diseases*, 18: 480–3. 22377052 10.3201/eid1803.111490 PMC3309593

[R23] —— et al. (2010) ‘An Outbreak of Highly Pathogenic H5N1 Avian Influenza in Korea, 2008’, *Veterinary Microbiology*, 141: 362–6. 19800184 10.1016/j.vetmic.2009.09.011

[R24] Kwon J. H. et al. (2020) ‘Domestic Ducks Play a Major Role in the Maintenance and Spread of H5N8 Highly Pathogenic Avian Influenza Viruses in South Korea’, *Transboundary and Emerging Diseases*, 67: 844–51. 31675474 10.1111/tbed.13406

[R25] —— et al. (2016) ‘Highly Pathogenic Avian Influenza A(H5N8) Viruses Reintroduced into South Korea by Migratory Waterfowl, 2014-2015’, *Emerging Infectious Diseases*, 22: 507–10. 26890406 10.3201/eid2203.151006 PMC4766904

[R26] —— et al. (2017) ‘Reassortant Clade 2.3.4.4 Avian Influenza A(H5N6) Virus in a Wild Mandarin Duck, South Korea, 2016’, *Emerging Infectious Diseases*, 23: 822–6. 28240976 10.3201/eid2305.161905 PMC5403023

[R27] Lee D. H. (2020) ‘Complete Genome Sequencing of Influenza A Viruses Using Next-Generation Sequencing’, *Methods in Molecular Biology (Clifton, N.J.)*, 2123: 69–79. 10.1007/978-1-0716-0346-8_6 32170681

[R28] Lee Y. J. et al. (2014) ‘Novel Reassortant Influenza A(H5N8) Viruses, South Korea, 2014’, *Emerging Infectious Diseases*, 20: 1087–9. 24856098 10.3201/eid2006.140233 PMC4036756

[R29] Liu J. H. et al. (2004) ‘Interregional Transmission of the Internal Protein Genes of H2 Influenza Virus in Migratory Ducks from North America to Eurasia’, *Virus Genes*, 29: 81–6. 15215686 10.1023/B:VIRU.0000032791.26573.f1

[R30] Makarova N. V. et al. (1999) ‘Transmission of Eurasian Avian H2 Influenza Virus to Shorebirds in North America’, *Journal of General Virology*, 80: 3167–71. 10.1099/0022-1317-80-12-3167 10567648

[R31] Mase M. et al. (2005) ‘Characterization of H5N1 Influenza A Viruses Isolated during the 2003-2004 Influenza Outbreaks in Japan’, *Virology*, 332: 167–76. 15661149 10.1016/j.virol.2004.11.016

[R32] Minin V. N., and Suchard M. A. (2008) ‘Fast, Accurate and Simulation-free Stochastic Mapping’, *Philosophical Transactions of the Royal Society**B: Biological Sciences*, 363: 3985–95. 10.1098/rstb.2008.0176 PMC2607419 18852111

[R33] Minin V. N., Bloomquist E. W., and Suchard M. A. (2008) ‘Smooth Skyride through a Rough Skyline: Bayesian Coalescent-based Inference of Population Dynamics’, *Molecular Biology and Evolution*, 25: 1459–71. 18408232 10.1093/molbev/msn090 PMC3302198

[R34] Piaggio A. J. et al. (2012) ‘Molecular Surveillance of Low Pathogenic Avian Influenza Viruses in Wild Birds across the United States: Inferences from the Hemagglutinin Gene’, *PLoS One*, 7: e50834. 10.1371/journal.pone.0050834 PMC3514193 23226543

[R35] Sakoda Y. et al. (2012) ‘Reintroduction of H5N1 Highly Pathogenic Avian Influenza Virus by Migratory Water Birds, Causing Poultry Outbreaks in the 2010-2011 Winter Season in Japan’, *Journal of General Virology*, 93: 541–50. 10.1099/vir.0.037572-0 22113008

[R36] Schafer J. R. et al. (1993) ‘Origin of the Pandemic 1957 H2 Influenza A Virus and the Persistence of Its Possible Progenitors in the Avian Reservoir’, *Virology*, 194: 781–8. 7684877 10.1006/viro.1993.1319

[R37] Senne D. A., Pedersen J. C., and Panigrahy B. (2005) ‘Live-bird Markets in the Northeastern United States: A Source of Avian Influenza in Commercial Poultry’, *Avian Influenza*, 8: 19–24.

[R38] Shepard S. S. et al. (2016) ‘Viral Deep Sequencing Needs an Adaptive Approach: IRMA, the Iterative Refinement Meta-assembler’, *BMC Genomics*, 17: 708. 10.1186/s12864-016-3030-6 PMC5011931 27595578

[R39] Sorrell E. M. et al. (2010) ‘A 27-amino-acid Deletion in the Neuraminidase Stalk Supports Replication of an Avian H2N2 Influenza A Virus in the Respiratory Tract of Chickens’, *Journal of Virology*, 84: 11831–40. 20826691 10.1128/JVI.01460-10 PMC2977859

[R40] Stallknecht D. E. et al. (1990) ‘Effects of pH, Temperature, and Salinity on Persistence of Avian Influenza Viruses in Water’, *Avian Diseases*, 34: 412–8. 2142421

[R41] Stamatakis A. (2014) ‘RAxML Version 8: A Tool for Phylogenetic Analysis and Post-analysis of Large Phylogenies’, *Bioinformatics*, 30: 1312–3. 24451623 10.1093/bioinformatics/btu033 PMC3998144

[R42] Uchida Y. et al. (2008) ‘Highly Pathogenic Avian Influenza Virus (H5N1) Isolated from Whooper Swans, Japan’, *Emerging Infectious Diseases*, 14: 1427–9. 18760011 10.3201/eid1409.080655 PMC2603097

[R43] USDA-APHIS . (2020), Prevention and Control of H5 and H7 Avian Influenza in the Live Bird Marketing System Uniform Standards for a StateFederal-Industry Cooperative Program.

[R44] Webster R. G. et al. (1992) ‘Evolution and Ecology of Influenza A Viruses’, *Microbiological Reviews*, 56: 152–79. 1579108 10.1128/mr.56.1.152-179.1992 PMC372859

[R45] Wee S.-H. et al. (2006) ‘Outbreaks of Highly Pathogenic Avian Influenza (H5N1) in the Republic of Korea in 2003/04’, *Veterinary Record*, 158: 341–4. 10.1136/vr.158.10.341 16531583

[R46] Youk S. S. et al. (2020) ‘Live Bird Markets as Evolutionary Epicentres of H9N2 Low Pathogenicity Avian Influenza Viruses in Korea’, *Emerging Microbes & Infections*, 9: 616–27. 32183621 10.1080/22221751.2020.1738903 PMC7144223

