# Supplementary figures and images for: Transmission dynamics of low pathogenicity avian influenza (H2N2) viruses in live bird markets of the Northeast United States of America, 2013–2019
Source: Virus Evol. 2022 Feb 9;8(1):veac009. doi: 10.1093/ve/veac009 (PMC9048936; doi:10.1093/ve/veac009)

A) PB2

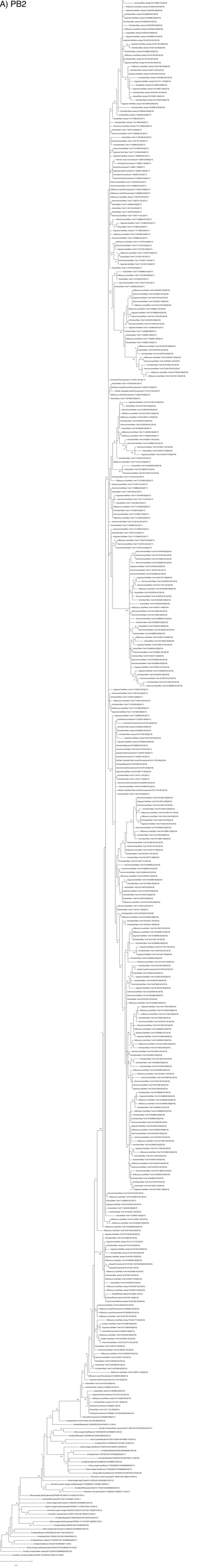



# C) PA

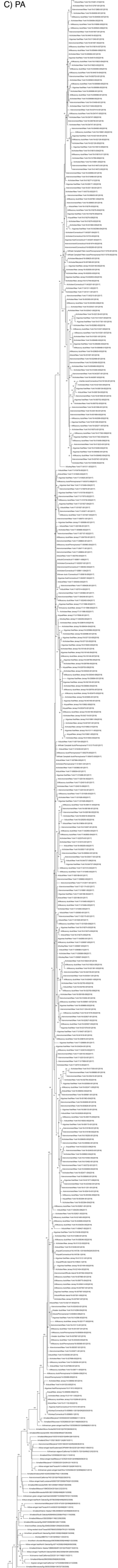



# E) NP

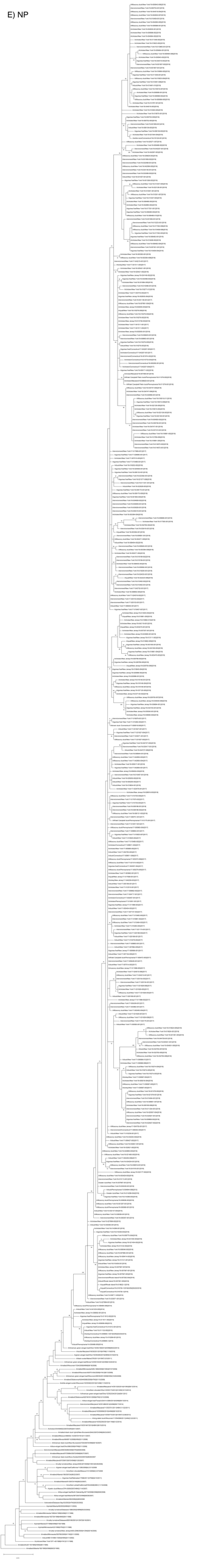

F) NA

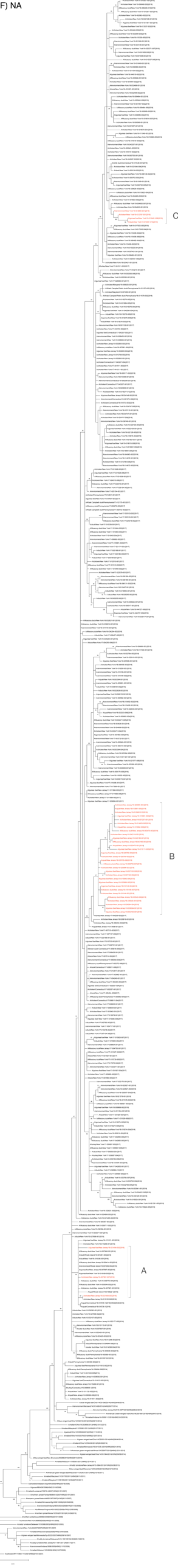



# H) NS

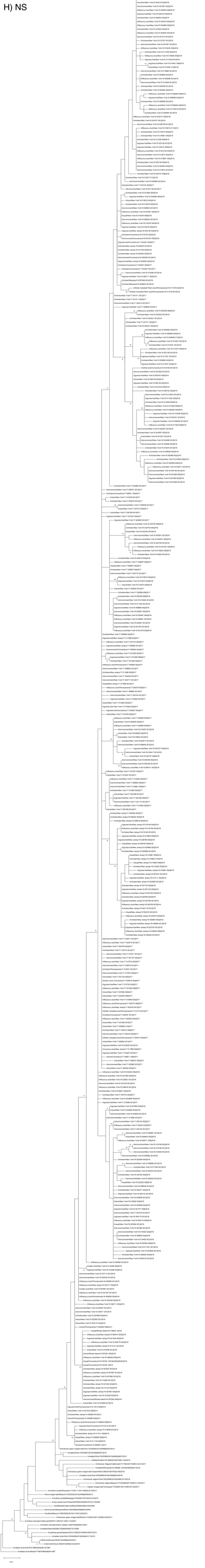

Supplement: veac009_Supp [file veac009_supp.zip › pdf_Supplemental_Figure1_8_gene_ML_NA_labeled.pdf]

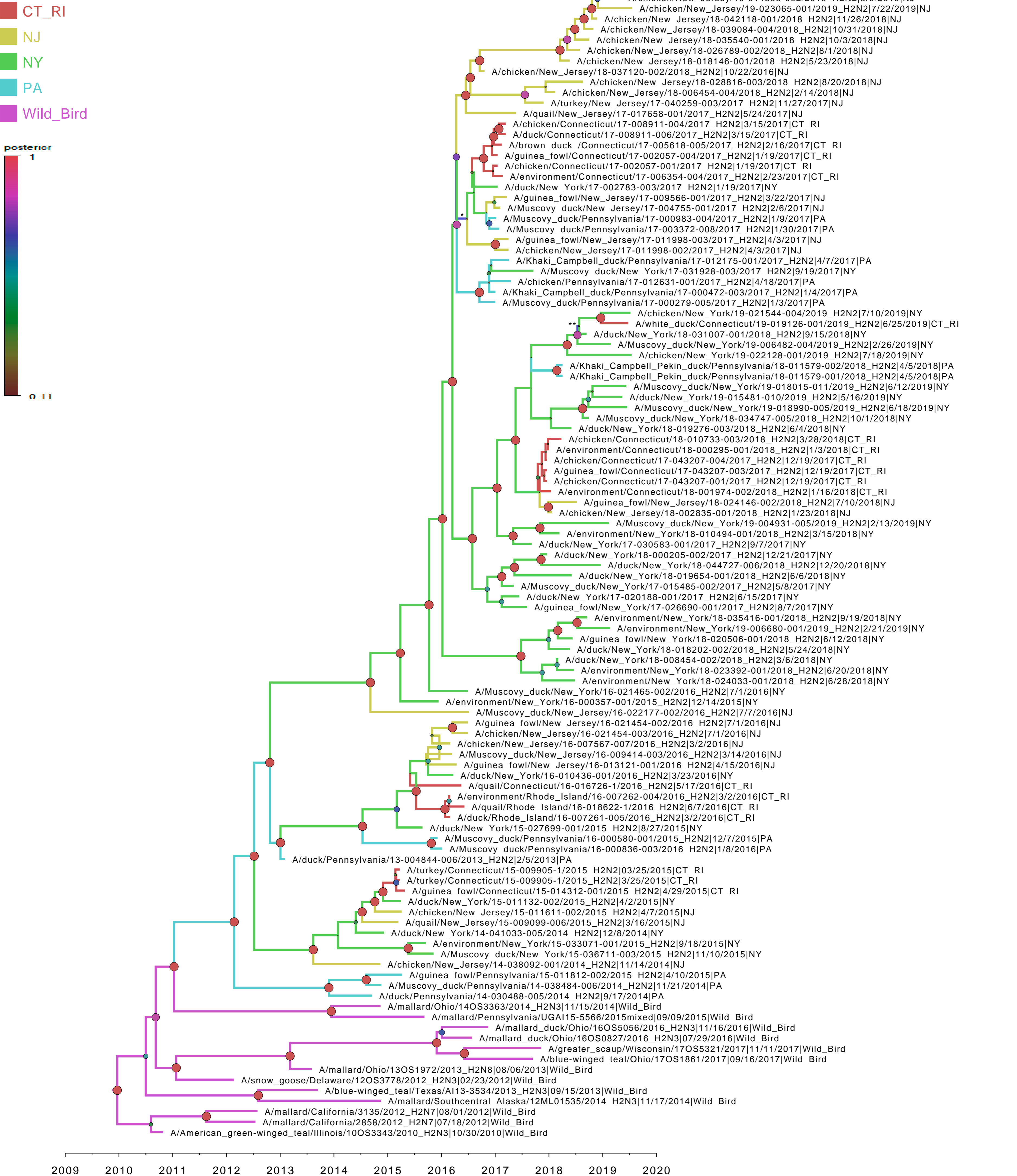

Supplement: veac009_Supp [file veac009_supp.zip › pdf_Supplemental_Figure2_Full_MCC_tree_Final.pdf]
